# Circulating GDF-15: a biomarker for metabolic dysregulation and aging in people living with HIV

**DOI:** 10.3389/fragi.2024.1414866

**Published:** 2024-06-04

**Authors:** Ling Wang, Juan Zhao, Madison Schank, Addison C. Hill, Puja Banik, Yi Zhang, Xiao Y. Wu, Janet W. Lightner, Shunbin Ning, Mohamed El Gazzar, Jonathan P. Moorman, Zhi Q. Yao

**Affiliations:** ^1^ Center of Excellence for Inflammation, Infectious Disease and Immunity, James H. Quillen College of Medicine, East Tennessee State University, Johnson City, TN, United States; ^2^ Department of Internal Medicine, Quillen College of Medicine, East Tennessee State University, Johnson City, TN, United States; ^3^ Hepatitis (HBV/HCV/HIV) Program, James H. Quillen VA Medical Center, Department of Veterans Affairs, Johnson City, TN, United States

**Keywords:** GDF-15, biomarker, metabolic dysregulation, aging, PLWH

## Abstract

Despite effective control of HIV replication by antiretroviral therapy (ART), a significant number of people living with HIV (PLWH) fail to achieve complete immune reconstitution and thus are deemed immune non-responders (INRs). Compared with immune responders (IRs) who have restored their CD4 T cell numbers and functions, CD4 T cells from these INRs exhibit prominent mitochondrial dysfunction and premature aging, which play a major role in increasing the incidence of non-AIDS, non-communicable diseases (NCDs). To date, there are no reliable biomarkers that can be used to typify and manage PLWH, especially INRs with non-AIDS NCDs. Growth differential factor-15 (GDF-15) is a transforming growth factor-β (TGF-β) family member known to regulate several biological processes involved in cell aging and stress responses. Since PLWH exhibit premature aging and metabolic dysregulation, here we measured the plasma levels of GDF-15 by ELISA and metabolic proteins by proteomic array and correlated the results with clinical parameters in ART-controlled PLWH (including INRs and IRs) and healthy subjects (HS). We found that GDF-15 levels were significantly elevated in PLWH compared to HS. GDF-15 levels were positively correlated with age and negatively associated with body mass and LDL cholesterol levels in the study subjects. Also, elevated GDF-15 levels were correlated with differential dysregulation of multiple metabolic proteins in PLWH. These results suggest that GDF-15 protein may serve as a biomarker of metabolic dysregulation and aging, and this biomarker will be useful in clinical trials targeting aging and metabolic disorders in ART-treated PLWH.

## Introduction

People living with HIV (PLWH) on antiretroviral therapy (ART) can achieve excellent virologic control, yet remain latently infected, and a significant number of PLWH fail to achieve complete immune reconstitution; these individuals are typified as immune non-responders (INRs) (Piconi et al., 2010; Lederman et al., 2011; Vidya Vijayan et al., 2017; Younes et al., 2018). Unlike immune responders (IRs) who usually restore their CD4 T cell numbers and functions, INRs persistently suffer from low CD4 T cell counts and poor cellular functions, exhibiting both immunologic scarring and low-grade inflammation (Kaufmann et al., 2005; Robbins et al., 2009; Shive et al., 2014; Nguyen et al., 2016), which lead to an immune aging phenotype (Nasi et al., 2017; Van Epps and Kalayjian, 2017). Indeed, we have previously reported that HIV infection can lead to premature CD4 T cell aging, as evidenced by overexpression of aging markers, shortened telomeres, and mitochondrial dysfunction (Zhao et al., 2019; Cao et al., 2020; Dang et al., 2020; Khanal et al., 2020; Nguyen et al., 2021; Zhao et al., 2021; Dang et al., 2022; Nguyen et al., 2022; Schank et al., 2023). This premature T cell aging exposes the host immune system to unique challenges that can induce immune derangements and increase the incidence of non-AIDS, non-communicable diseases (NCDs), such as cardiovascular diseases (CVD), non-AIDS-defining cancers, HIV-associated neurocognitive disorders (HAND), and all-cause morbidity and mortality (Baker et al., 2008; Carbone et al., 2014; Zanni et al., 2014; Watkins and Treisman, 2015; Patel et al., 2018; Shah et al., 2018). Classifying biomarkers associated with immune aging and metabolic dysregulation during latent HIV infection may identify therapeutic targets for PLWH, especially those INRs with non-AIDS NCDs.

Growth differential factor-15 (GDF-15), also known as macrophage inhibitory cytokine-1 (MIC-1), is a secreted ligand of the transforming growth factor-β (TGF-β) superfamily receptors. Interactions of GDF-15 with TGF-β receptors expressed on a broad range of cell types lead to recruitment and activation of SMAD transcription factors that regulate gene expression of pleiotropic proteins involved in several biological processes, including cell aging and stress responses (Wischhusen et al., 2020). GDF-15 expression is often induced under stress conditions to maintain cell and tissue homeostasis. Increased GDF-15 levels are associated with pathologic conditions such as inflammation, infection, tissue injury, and liver, kidney, cardiovascular diseases, and cancers (Wischhusen et al., 2020; Delrue et al., 2023; Losch et al., 2023; Merchant et al., 2023; Morita-Tanaka et al., 2023; Teawtrakul et al., 2023). GDF-15 has thus been widely explored as a biomarker for disease prognosis. Recent studies reported that GDF15 levels positively correlated with age, and negatively correlated with telomere length, telomerase activity, and hTERT mRNA (Liu et al., 2021); knockdown of GDF15 protein induces mitochondrial dysfunction and premature senescence in human dermal fibroblasts (Wedel et al., 2023). The strong predictive value of GDF-15 as a biomarker may plausibly be linked to its role in aging and immunometabolism.

Since PLWH exhibit an immune aging phenotype and increased incidence of non-AIDS NCDs with metabolic disorders, in this study we assessed changes in plasma levels of GDF-15 and metabolic proteins. Our results demonstrate that increases in GDF-15 levels are associated with immuno-metabolic dysregulation and aging in PLWH, especially in HIV-INRs.

## Results

### Circulating GDF-15 protein is elevated in ART-treated PLWH and closely correlates with age

**FIGURE 1:**
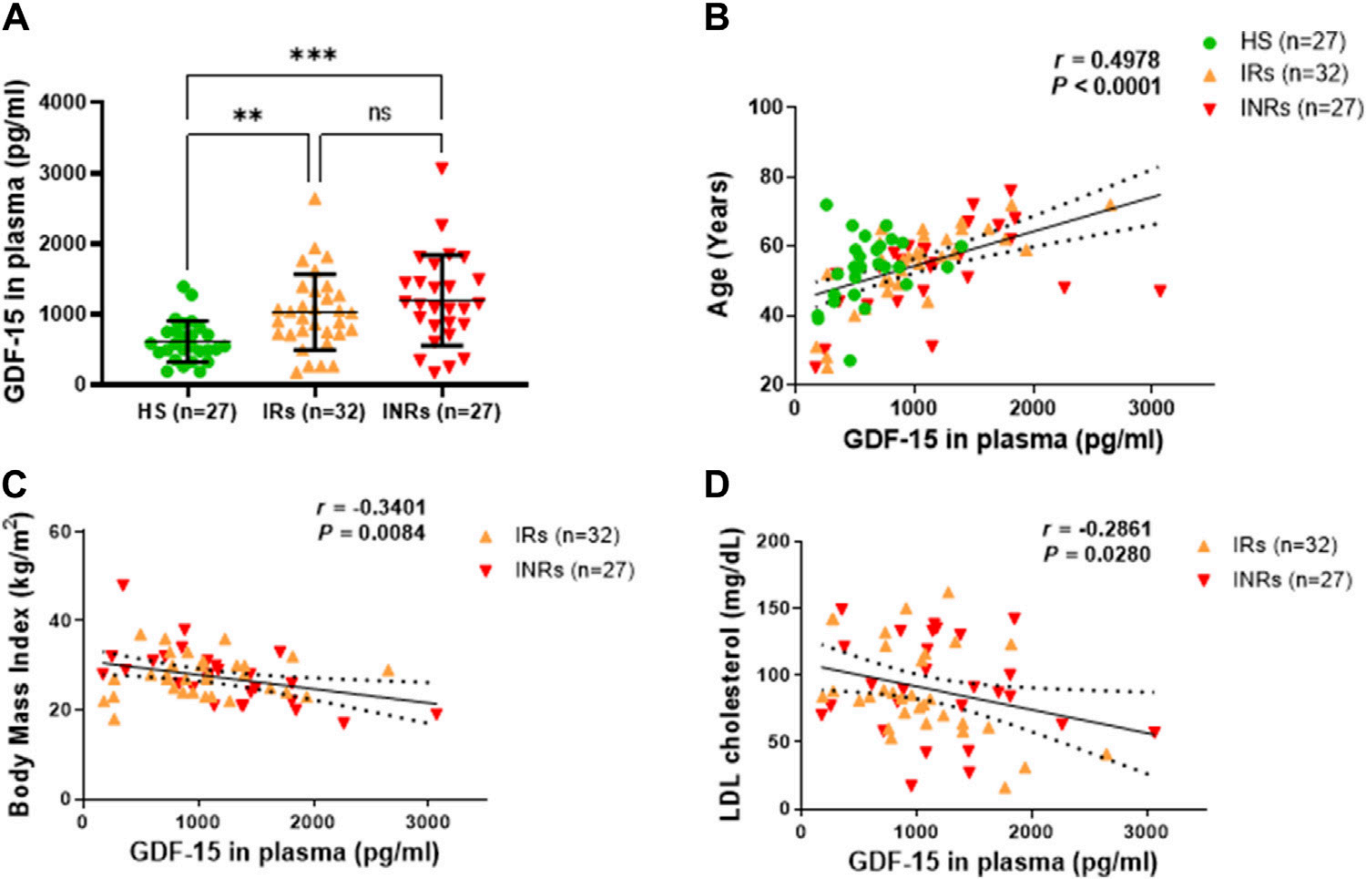
GDF-15 protein is elevated and associated with age, body mass, and LDL cholesterol levels in PLWH. **(A)** The plasma levels of GDF-15 in HIV-IRs, HIV-INRs, and HS, measured by ELISA as described in Materials and Methods. **(B–D)** Correlation of circulating GDF-15 levels with age, body mass index, and LDL cholesterol levels in the study subjects. HS, healthy subjects; IRs, immune responders; INRs, immune non-responders. NS, not significant; *, significant; **, ***, ****, very significant.

Because GDF-15 is deemed a marker of cellular aging and mitochondrial stress, and PLWH exhibit premature aging and metabolic disorders, we measured plasma levels of GDF-15 in PLWH (including IRs and INRs) and healthy subjects (HS) by ELISA. As shown in Figure 1A, circulating GDF-15 protein was elevated in PLWH, with higher levels in INRs (*n* = 27) and IRs (*n* = 32) compared to HS (*n* = 27). Notably, elevated GDF-15 levels positively correlated with age (*p* < 0.0001), which matched in all three groups of subjects (HIV-INRs, HIV-IRs, and HS) (Figure 1B and Supplementary Figure S1A). In addition, GDF-15 levels were negatively associated with the body mass index (*p* = 0.0084, Figure 1C) and low-density lipoprotein (LDL) cholesterol levels (*p* = 0.0280, Figure 1D) in PLWH, likely due to the notion that aging PLWH tend to have lower body mass and LDL cholesterol levels. We did not observe any correlation between the increases in GDF15 levels and peripheral blood CD4 T cell count (Supplementary Figure S1B), nadir CD4 count (Supplementary Figure S1C), baseline viral load prior to ART (Supplementary Figure S1D), years since HIV diagnosis (Supplementary Figure S1E), years on ART (Supplementary Figure S1F), levels of triglycerides (Supplementary Figure S1G), total cholesterol (Supplementary Figure S1H), HDL cholesterol (Supplementary Figure S1H), hemoglobin A1c (Supplementary Figure S1I), systolic blood pressure (Supplementary Figure S1J), or diastolic blood pressure (Supplementary Figure S1K) in INRs and IRs. Also, there were no differences in the GDF-15 levels between male (*n* = 46) and female (*n* = 12) patients (Supplementary Figure S2A); smoking status (smoking vs. nonsmoking, *n* = 15 and *n* = 44, respectively) (Supplementary Figure S2B); patients with (n = 38) and without (*n* = 20) hypertension (Supplementary Figure S2C); and patients with (*n* = 14) and without diabetes (*n* = 45) (Supplementary Figure S2D). Moreover, we did not observe any differences in GDF-15 levels between patients who received different ART regimens (Supplementary Figure S2E). These results demonstrate a significant increase in GDF-15 protein in PLWH that is correlated positively with age and negatively with body mass and LDL cholesterol levels.

### Elevated GDF-15 levels correlate with metabolic proteins that are increased in HIV-INRs and/or HIV-IRs

**FIGURE 2:**
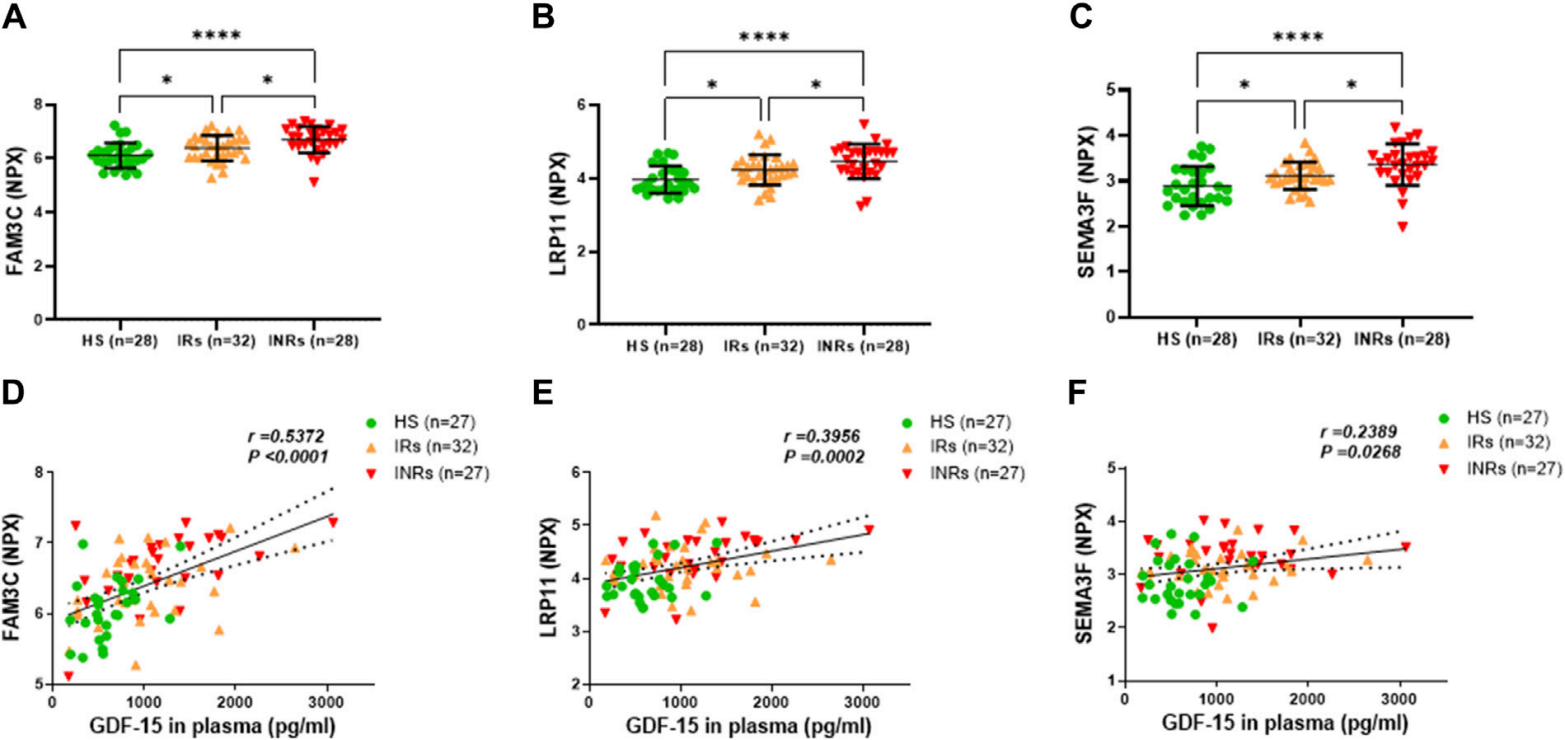
Metabolic proteins upregulated in HIV-INRs and HIV-IRs and their correlations with plasma GDF-15 levels. **(A–C)** Levels of FAM3C, LRP11 and SEMA3F proteins in plasma from HIV-INRs. HIV-IRs, and HS, measured by Olink proteomic array with metabolism panel and proximity extension assay. The results are presented as a normalized protein expression (NPX) value per Olink Proteomics’ arbitrary unit on a log2 scale. **(D–F)** Correlation of FAM3C, LRP11, and SEMA3F proteins with GDF-15 levels. *n* = number of subjects.

**FIGURE 3:**
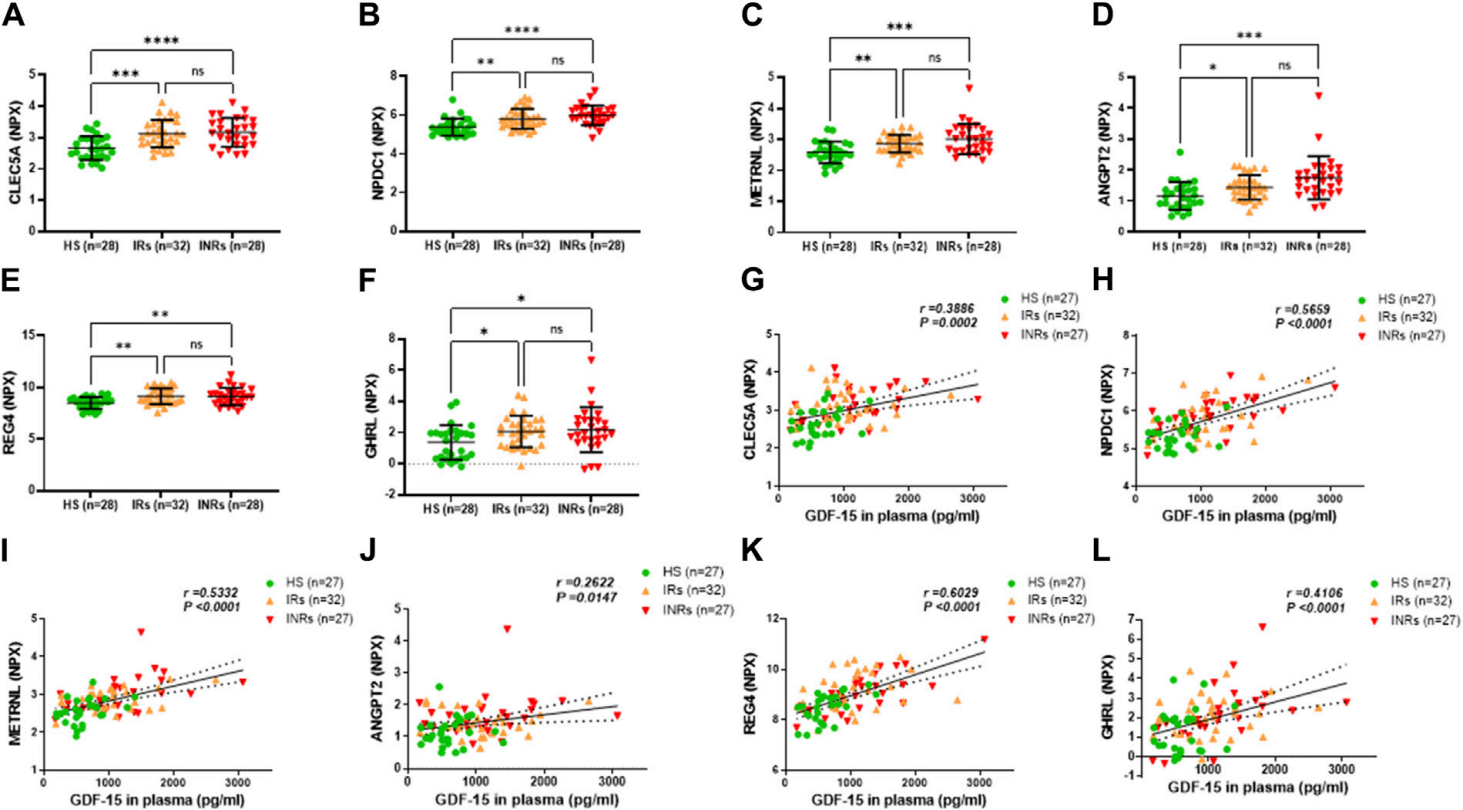
Metabolic proteins upregulated in HIV-INRs and HIV-IRs and their correlations with GDF-15 levels. **(A–F)** Levels of CLEC5A, NPDC1, METRNL, ANGPT2, REG4, and GHRL proteins in plasma from HIV-INRs, HIV-IRs, and HS, measured by Olink proteomic array. **(G–L)** Correlation of CLEC5A, NPDC1, METRNL, ANGPT2, REG4, and GHRL proteins with GDF-15 levels. n = number of subjects.

**FIGURE 4:**
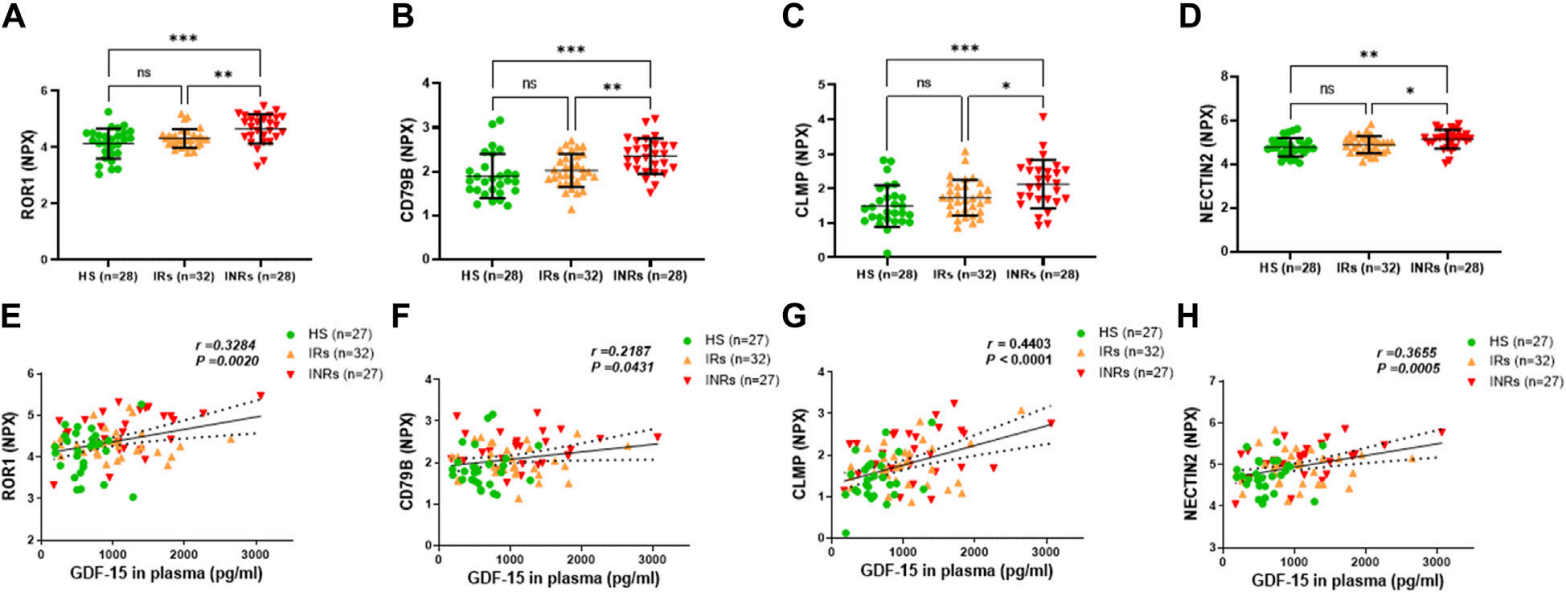
Metabolic proteins upregulated in HIV-INRs and their correlations with GDF-15 levels. **(A–D)** Levels of ROR1, CD79B, CLMP, and NECTIN2 proteins in plasma from HIV-INRs, HIV-IRs, and HS, measured by Olink proteomic array. **(E–H)** Correlation of ROR1, CD79B, CLMP, and NECTIN2 proteins with GDF-15 levels. n = number of subjects.

**FIGURE 5:**
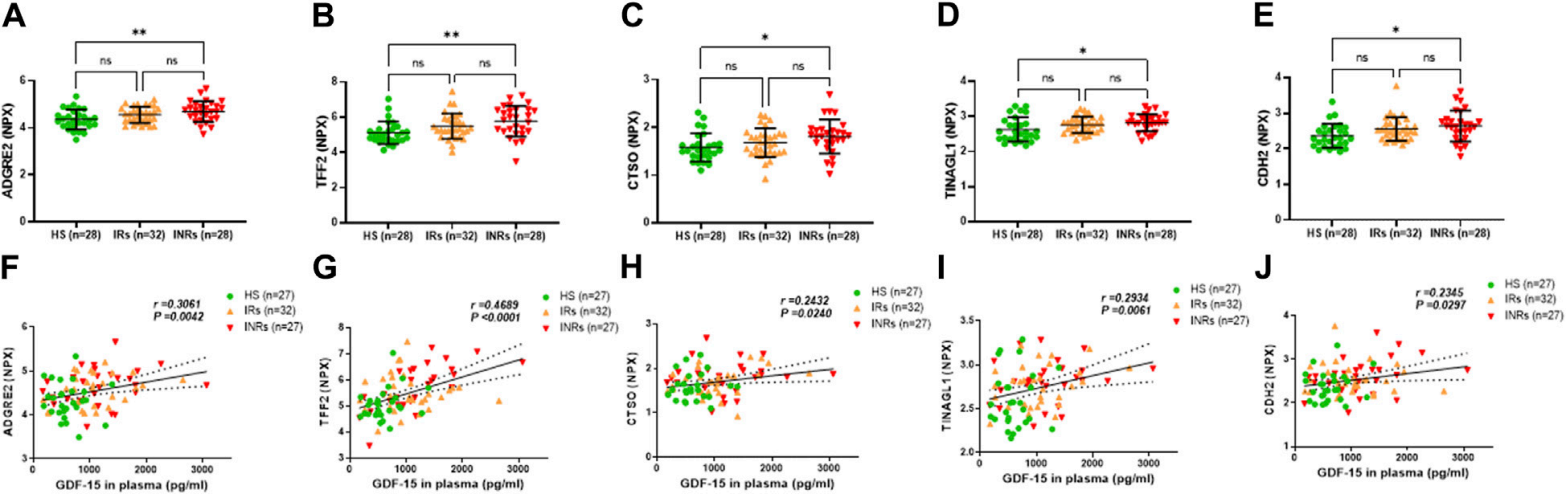
Metabolic proteins upregulated in HIV-INRs and their correlations with GDF-15 levels. **(A–E)** Levels of ADGRE2, TFF2, CTSO, TINAGL1, and CDH2 proteins in plasma from HIV-INRs, HIV-IRs, and HS, measured by Olink proteomic array. **(F–J)** Correlation of ADGRE2, TFF2, CTSO, TINAGL1, and CDH2 proteins with GDF-15 levels. n = number of subjects.

**FIGURE 6:**
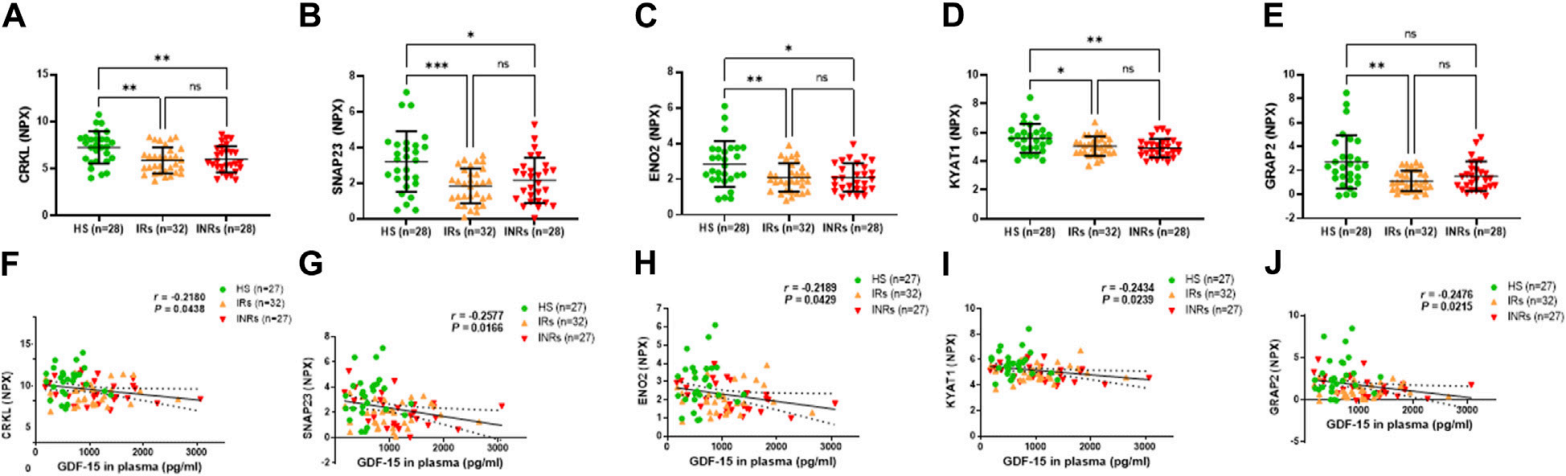
Metabolic proteins downregulated in HIV-IRs and/or HIV-INRs, and their correlations with GDF-15 levels. **(A–E)** Levels of CRKL, SNAP23, ENO2, KYAT1, and GRAP2 proteins in plasma from HIV-INRs, HIV-IRs, and HS, measured by Olink proteomic array. **(F–J)** Correlation of CRKL, SNAP23, ENO2, KYAT1, and GRAP2 proteins with GDF-15 levels. n = number of subjects.

Since PLWH exhibit metabolic disorders, we measured the plasma levels of 92 metabolic proteins using the Olink proteomic assay (metabolic panel) and then correlated changes in their levels with the circulating GDF-15 protein in the same subjects. We identified 34 metabolic proteins that positively or negatively correlated with the GDF-15 levels. Amongst these proteins, 9 proteins positively correlated with GDF-15 levels but demonstrated no significant differences between IRs, INRs and HS (Supplementary Figures S3A–R); 2 proteins were at similar levels in HIV-INRs, HIV-IRs, and HS but exhibited a negative correlation with GDF-15 levels (Supplementary Figures S3S–V); 23 proteins were significantly dysregulated in PLWH and divided into 6 groups based on changes in their plasma levels, i.e., normalized protein expression (NPX), in HIV-INRs, HIV-IRs, and HS groups. (Figures 2–6; Table 1).

**TABLE 1:** Demographics of the study subjects.

	Group 1	Group 2	Group 3	Group 4	Group 5	Group 6
Protein name	FAM3C, LRP11, SEMA3F	CLEC5A, NPDC1, METRNL, ANGPT2, REG4, GHRL	ROR1, CD79B, CLMP, NECTIN2	ADGRE2, TFF2, CTSO, TINAGL1, CDH2	CRKL, SNAP23, ENO2, KYAT1	GRAP2
Correlated with GDF-15	Positively	Positively	Positively	Positively	Negatively	Negatively
INRs vs. IRs	Increased	No difference	Increased	No difference	No difference	No difference
INRs vs. HS	Increased	Increased	Increased	Increased	Decreased	No difference
IRs vs. HS	Increased	Increased	No difference	No difference	Decreased	Decreased

Group 1 included metabolic proteins that were upregulated in both HIV-INRs and HIV-IRs, showed significant differences in NPX levels amongst INRs, HIV-IRs, and HS, and in which their levels positively correlated with the GDF-15 levels (Figure 2; Table 1). Specifically, the NPX levels of the family with sequence similarity 3 member C (FAM3C) protein, low-density lipoprotein receptor-related protein 11 (LRP11), and semaphorin-3F (SEMA3F) protein were significantly elevated in PLWH, especially in HIV-INRs (n = 28), compared to HS (*n* = 28) (Figures 2A–C). Notably, these proteins were also significantly higher in INRs compared to IRs. Moreover, the increase in NPX levels of plasma FAM3C, LRP11, and SEMA3F proteins positively correlated with the elevated GDF-15 levels in these subjects (Figures 2D–F).

Group 2 included metabolic proteins that were significantly upregulated in both HIV-INRs and HIV-IRs compared to HS but with no significant differences between HIV-INRs and HIV-IRs (Figure 3; Table 1). As shown in Figures 3A–F, NPX levels of the C-type lectin member 5A (CLEC5A), neural proliferation differentiation and control protein 1 (NPDC1), meteorin-like/meteorin-beta/IL-41 (METRNL), angiopoietin-2 (ANGPT2), regenerating islet-derived 4 (REG4), and ghrelin (GHRL) proteins were increased in the plasma of both HIV-INRs and HIV-IRs compared to HS. Also, the increases in these metabolic proteins positively correlated with the levels of GDF-15 in these subjects (Figures 3G–L).

Group 3 comprised metabolic proteins that were significantly upregulated only in HIV-INRs compared to HIV-IRs and HS (Figure 4; Table 1). These proteins included receptor tyrosine-like orphan receptor 1 (ROR1), cluster of differentiation 79 B (CD79B), CXADR-like membrane protein (CLMP), and nectin cell adhesion molecule 2 (NECTIN2) (Figures 4A–F). Notably, these upregulated metabolic proteins also positively correlated with the elevated GDF-15 levels in the study subjects (Figures 4E–H).

Group 4 comprised metabolic proteins that were significantly upregulated only in HIV-INRs compared to HS (Figure 5; Table 1). These proteins included adhesion G protein-coupled receptor E2 (ADGRE2), trefoil factor 2 (TFF2), cathepsin O (CTSO), tubulointerstitial nephritis antigen-like 1 (TINAGL1), and cadherin 2 (CDH2) (Figures 5A–F). Notably, levels of these metabolic proteins also positively correlated with the circulating GDF-15 protein in the study subjects (Figures 5F–J).

### Metabolic proteins that are decreased in HIV-INRs and/or HIV-IRs and negatively correlate with GDF-15 levels

Groups 5 and 6 comprised metabolic proteins that negatively correlated with circulating GDF-15 levels (Figure 6; Table 1). The metabolic proteins in Group 5 were decreased in HIV-INRs and HIV-IRs compared to HS, including crk-like protein (CRKL), synaptosomal-associated protein (SNAP23), enolase 2 (ENO2), and kynurenine-oxoglutarate transaminase 1 (KYAT1) (Figures 6A–D). The GRB2-related adapter protein 2 (GRAP2) is the only metabolic protein in Group 6 that was downregulated only in HIV-IRs compared to HS (Figure 6E).

## Discussion

HIV-INR patients fail to achieve complete immune reconstitution despite virological control by ART, the molecular mechanisms underlying immuno-metabolic dysregulation in HIV-INRs remain elusive. Additionally, biomarkers to typify PLWH are lacking and there are no specific molecules that can be targeted for immunotherapy. A recent study reported elevated levels of the mitochondrial stress marker GDF-15 in PLWH that were associated with aging, HIV reservoir size, and increased risk of developing non-AIDS comorbidities (Isnard et al., 2021). In our study, we found that elevated GDF-15 levels in ART-controlled PLWH correlated with age, body mass index, LDL cholesterol levels and multiple metabolic proteins that were differentially regulated in PLWH, especially in HIV-INRs. These results indicate that circulating GDF-15 might serve as a biomarker of metabolic dysregulation and aging in PLWH on ART.

Because this study focuses on immuno-metabolic derangements in HIV-INRs, here we only discuss metabolic proteins that are significantly dysregulated in HIV-INRs and correlate with the GDF-15 levels. Specifically, FAM3C, LRP11, and SEMA3F were significantly increased in HIV-INRs compared to HIV-IRs. In addition, ROR1, CD79B, CLMP, and NECTIN2 proteins were elevated in HIV-INRs compared to HIV-IRs and HS. Notably, these proteins have been implicated in several biological processes related to mitochondrial stress responses, especially in the setting of cancers. For example, recent studies have shown that increases in FAM3C protein levels are closely associated with tumor formation, invasion, metastasis, and poor survival, suggesting that FAM3C may serve as a potential biomarker and therapeutic target in cancer (Zhu et al., 2021). It has been reported that LRP11 activates β-catenin to induce PD-L1 expression in prostate cancer and that a high LRP11 level positively correlates with PD-L1 expression in cancer tissue (Gan et al., 2020). SEMA3F has been implicated in immune signaling and immune synapse formation, and in regulating the localization and retention of tumor-associated macrophages (Lou et al., 2021). RoR1 is an embryonic protein that is only detectable in embryonic tissue and generally absent in adult tissue (Borcherding et al., 2014). While the mechanism driving RoR1 expression in PLWH is unclear, upregulation of ROR1 (as well as other metabolic proteins) in HIV-INRs suggests that these proteins can be a potential target for immunotherapy.

Several metabolic proteins in which we observed a differential dysregulation in PLWH have been implicated in cell signaling or metabolic processes. CD79 is a transmembrane protein that forms a complex with the B-cell receptor (BCR) for cell signaling following antigen recognition by the BCR (Chu and Arber, 2001). CD79 is composed of two distinct chains (CD79A and CD79B) and each chain contains an immunoreceptor tyrosine-based activation motif (ITAM) in their intracellular tails for B cell signaling, similar to the CD3-generated signal transduction observed during T cell receptor activation on T cells (Chu and Arber, 2001). CLMP protein is expressed and localized to the junction complexes that are formed between endothelial and epithelial cells and may play a role in cell-cell adhesion, and expression of CLMP in white adipose NECTIN2 (or PVRL2) is a cell adhesion molecule involved in lipid metabolism, it is an important marker for progressive carotid atherosclerosis (Li et al., 2021). Notably, NECTIN2 is significantly decreased in response to plasma cholesterol reduction (Rossignoli et al., 2017). Previous studies demonstrated that the downregulation of NECTIN2 by siRNA led to an increase in LDL cholesterol uptake in cells (Blattmann et al., 2013), and NECTIN2 knockout mice showed a reduction in atherosclerosis (Rossignoli et al., 2017). Both studies indicated a reduction in LDL cholesterol upon downregulation of NECTIN2. The mechanism by which NECTIN2 regulates LDL cholesterol levels remains unclear. One plausible hypothesis suggests that NECTIN2 may interact with cell surface receptors or trigger intracellular signaling pathways involved in the uptake or processing of LDL cholesterol particles by cells. In our study, we observed a positive correlation between NECTIN2 and GDF15 levels. However, LDL cholesterol exhibited a negative correlation with GDF15 levels. This suggests that LDL cholesterol may be regulated by alternative pathways in PLWH. In addition to the proteins that were upregulated in HIV-INRs and/or HIV-IRs and positively associated with elevated GDF-15 levels, our results revealed a group of metabolic proteins that were significantly downregulated in PLWH and negatively correlated with circulating GDF-15 levels. The significant changes in these metabolic proteins may provide an important link between immune aging and immunometabolic dysregulation in PLWH, especially in HIV-INRs with non-AIDS NCDs, which warrants further studies.

Employing a well-matched cohort of patients, this study provided fairly clear evidence that GDF15 is elevated in HIV-infected subjects, whether IRs or INRs. Given these individuals have robust virologic control, circulating virus would appear not to be at the core of this aberration, and what is driving upregulation of GDF15 requires further study. While it remains possible that ART itself might initiate this, our data (Supplementary Figure S2E) showing no difference in GDF15 levels with any given ART regimen argues against this. It is also feasible that low-grade production of viral products (RNAs and/or proteins) generated from the HIV reservoir or a low-grade inflammatory and tissue/cell injury process might play a role. Further mechanistic studies in elucidating the interrelationship between GDF-15 and each individual metabolic protein are clearly warranted to understand their role in the development and management of non-AIDS NCDs in PLWH on ART.

In summary, in this study, we found that circulating GDF-15 levels were significantly elevated in PLWH compared with HS. Elevated GDF-15 levels correlated positively with age and negatively with body mass and LDL cholesterol levels. Also, the increases in GDF-15 levels correlated with changes in multiple metabolic proteins that were differentially dysregulated during latent HIV infection. Notably, there are several unexplored aspects in this study, such as risk factors and the mechanisms underlying the increases in GDF-15 and dysregulations of specific metabolic proteins. Such underlying factors/mechanisms may include specific pathophysiological triggers, cell types, and signaling pathways, which warrant further investigations. Due to the heterogenous of our patient cohort and limited numbers in this study, our findings showed highly overlapping data between each patient groups. In future studies, a larger sample size will be employed to mitigate the impact of subject heterogeneity across various clinical parameters, such as age, gender, race, BMI, HIV infection and treatment history. This expanded cohort will also allow for a more comprehensive assessment of the stability and reproducibility of the data obtained in the study and address these issues computationally. Nevertheless, our results indicate that circulating GDF-15 protein can serve as a biomarker for aging and immuno-metabolic dysregulation in PLWH on ART. Thus, assessing the levels of GDF-15 and associated metabolic proteins can be useful in clinical trials targeting aging and metabolic disorders in PLWH on ART.

## Materials and methods

### Study subjects

The study protocol was approved by the joint Institutional Review Board (IRB) of East Tennessee State University and James H. Quillen VA Medical Center (ETSU/VA IRB# 0519.24s). Written informed consent was obtained from all participants. The study included two populations: 60 PLHIV on ART with undetectable viremia (HIV-RNA <20 copies/mL), consisting of 32 HIV-IRs (>500 CD4 T cells/ul) and 28 HIV-INRs (<500 CD4 T cells/ul); and 28 age and gender-matched healthy subjects (HS, blood obtained from BioIVT, Gray, TN). Subjects with malignancy, transplantation, HBV or HCV infection, or immunosuppressive drug treatment were excluded. Fresh plasma from whole blood samples was collected and stored at −80°C. The characteristics of the study subjects are shown in Table 2.

**TABLE 2:** Metabolic proteins that are dysregulated in PLWH and associated with GDF-15 levels.

Subjects	Number	Sex	Median age	Median CD4 T cell count
HS	28	24M/4F	54 (27–72)	N/A
HIV-IR	32	28M/4F	57 (25–72)	885 (501–1352)
HIV-INR	28	24M/4F	56 (25–76)	358 (28–469)

### Olink proteomics analysis

Olink^®^ Target 96 Metabolism panel offers a broad selection of proteins involved in metabolic regulation. Excluding the assay control, levels of 92 metabolic proteins were simultaneously measured in the plasma from HS and PLWHIV by the proximity extension assay (PEA). The assay results are presented as a normalized protein expression (NPX) value per Olink Proteomics’ arbitrary unit on a log2 scale.

### Enzyme-linked immunosorbent assay (ELISA)

The human GDF-15 Quantikine ELISA Kit (R&D Systems, Minneapolis, MN) was used to determine GDF-15 levels in the plasma of PLWH and HS according to the manufacturer’s instructions. This 96-well strip plate, solid phase sandwich ELISA kit was designed to measure GDF-15 in cell culture supernatants, serum, plasma, and urine, with great sensitivity (minimum 4.39 pg/mL, preferred assay range 23.4–1,500 pg/mL), specificity (<0.5% cross-reactivity and no significant interference observed with available related molecules). Results obtained using natural GDF-15 showed linear curves that were parallel to the standard curves obtained using the kit standards.

### Statistics

The data were analyzed using Prism 9.3 software (GraphPad Software, San Diego, CA) and are presented as mean ± SD. Correlations were made by Pearson’s correlation. Welch’s correction was utilized if unequal variances were found. Comparisons between three groups were analyzed by One-way ANOVA after excluding outliers that were identified by the ROUT method (Q = 1.000%). Statistical significance is reported with *p < 0.05; **p < 0.01; ***p < 0.001 and ****p < 0.0001.

## Data Availability

The raw data supporting the conclusion of this article are available from the corresponding author on reasonable request.
