# Supplementary material for: Circulating GDF-15: a biomarker for metabolic dysregulation and aging in people living with HIV
Source: Front Aging. 2024 Jun 4;5:1414866. doi: 10.3389/fragi.2024.1414866 (PMC11183798; doi:10.3389/fragi.2024.1414866)
Supplement: Supplementary file 1 [file Presentation1.PPTX]

## Slide 1
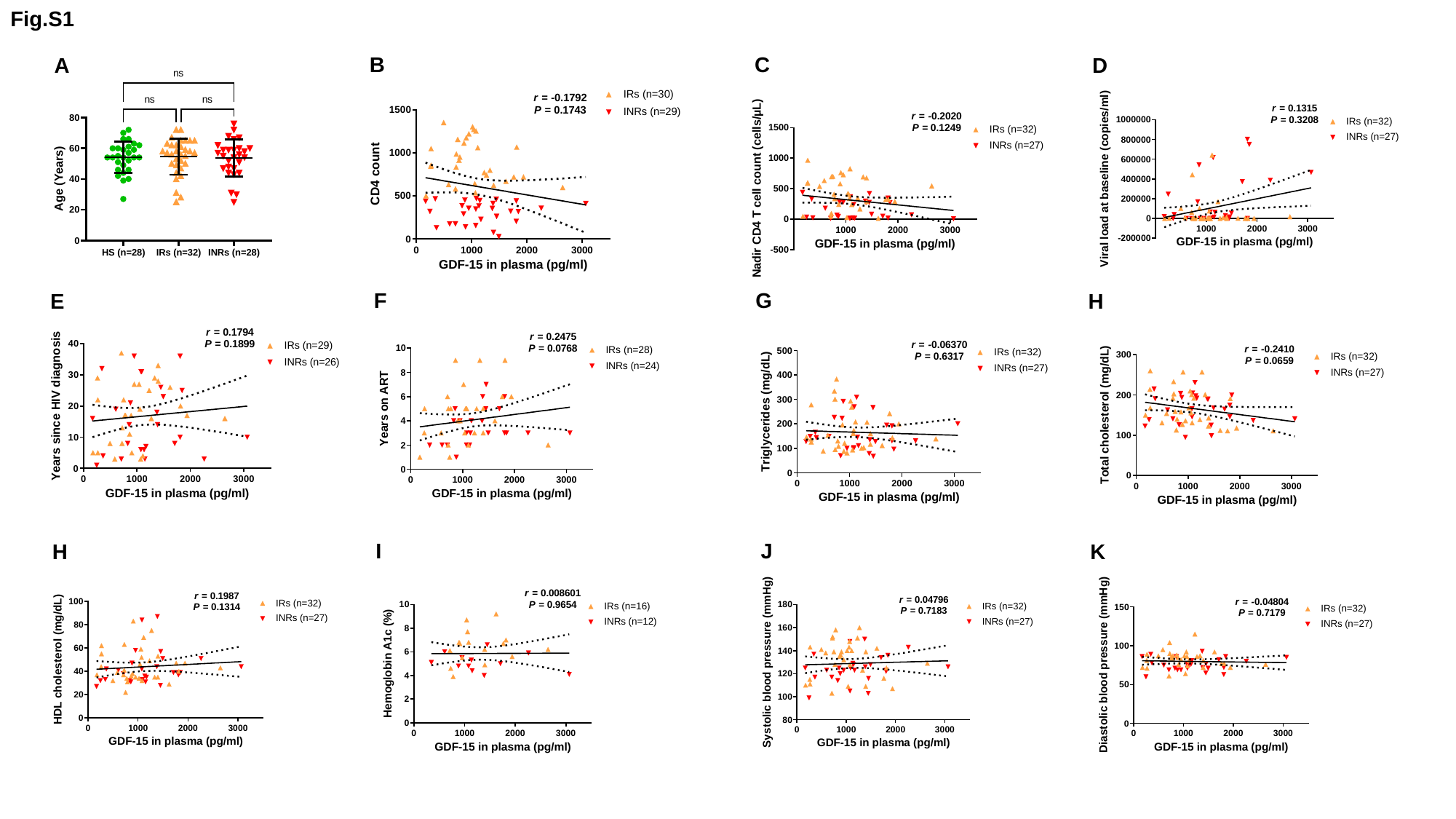

Fig.S1
C
B
D
A
G
F
H
E
J
I
K
H

## Slide 2
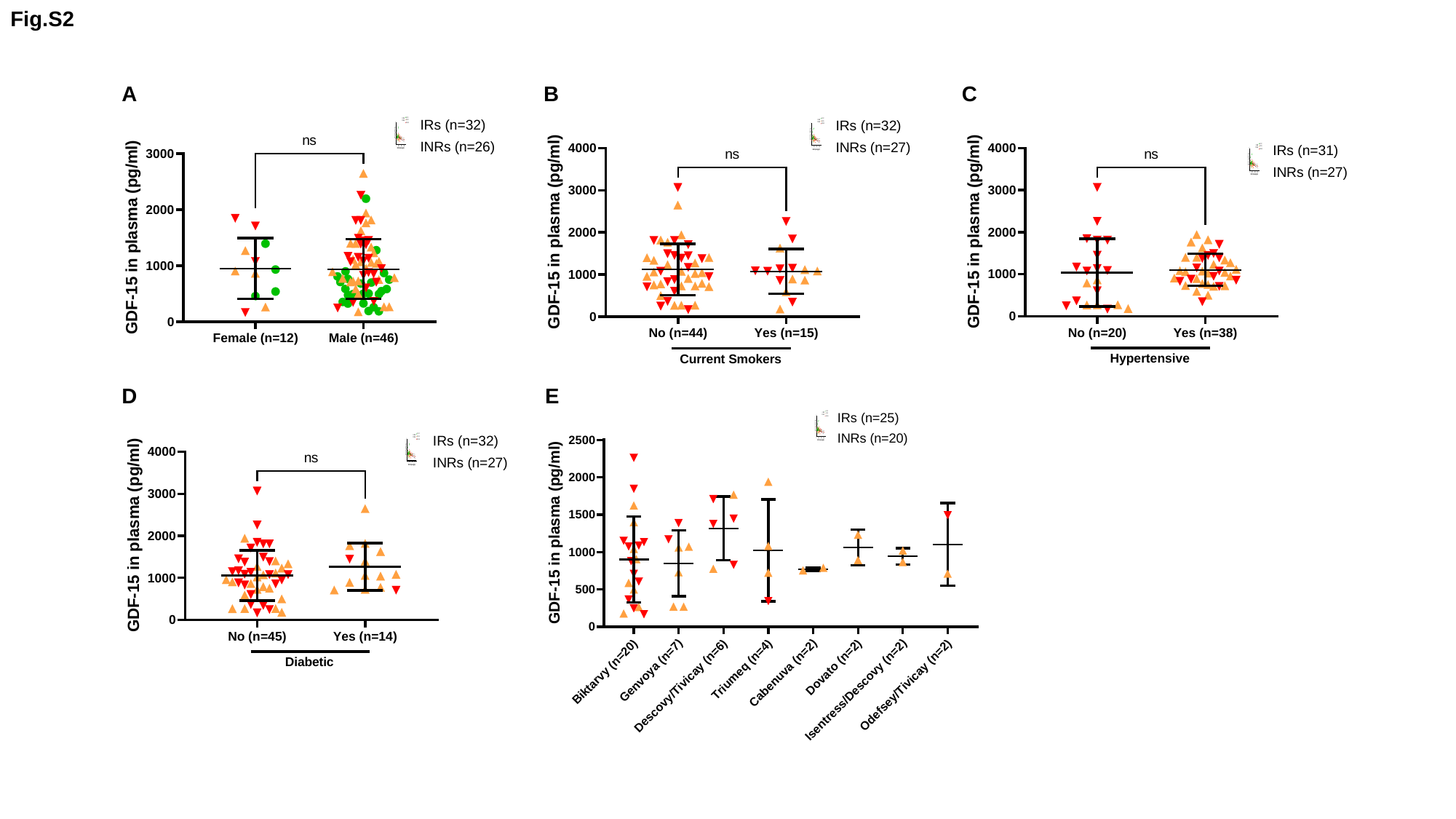

Fig.S2
C
B
A
D
E

## Slide 3
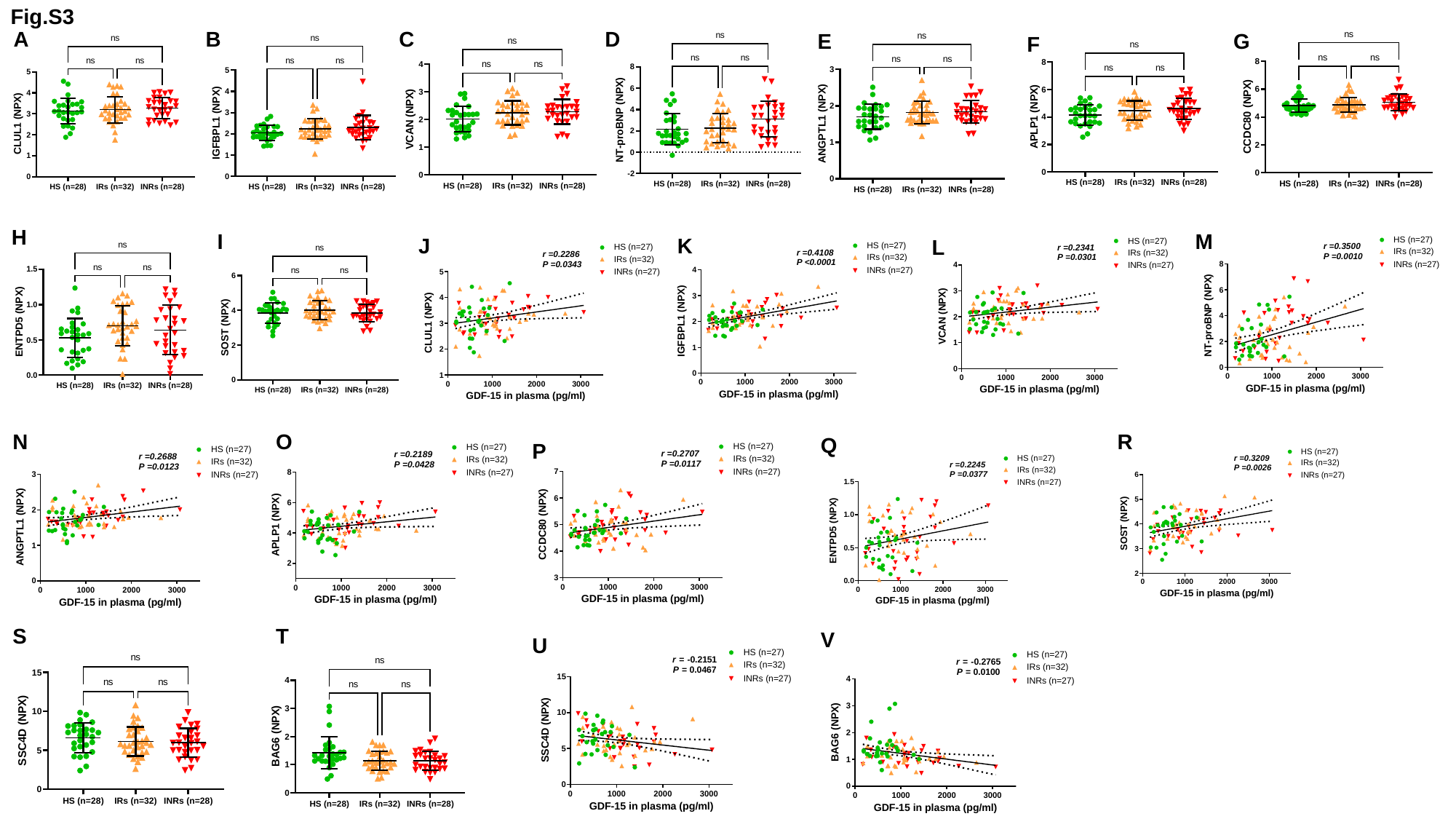

Fig.S3
D
A
B
C
G
E
F
H
I
M
J
K
L
R
N
O
Q
P
S
T
V
U

## Slide 4
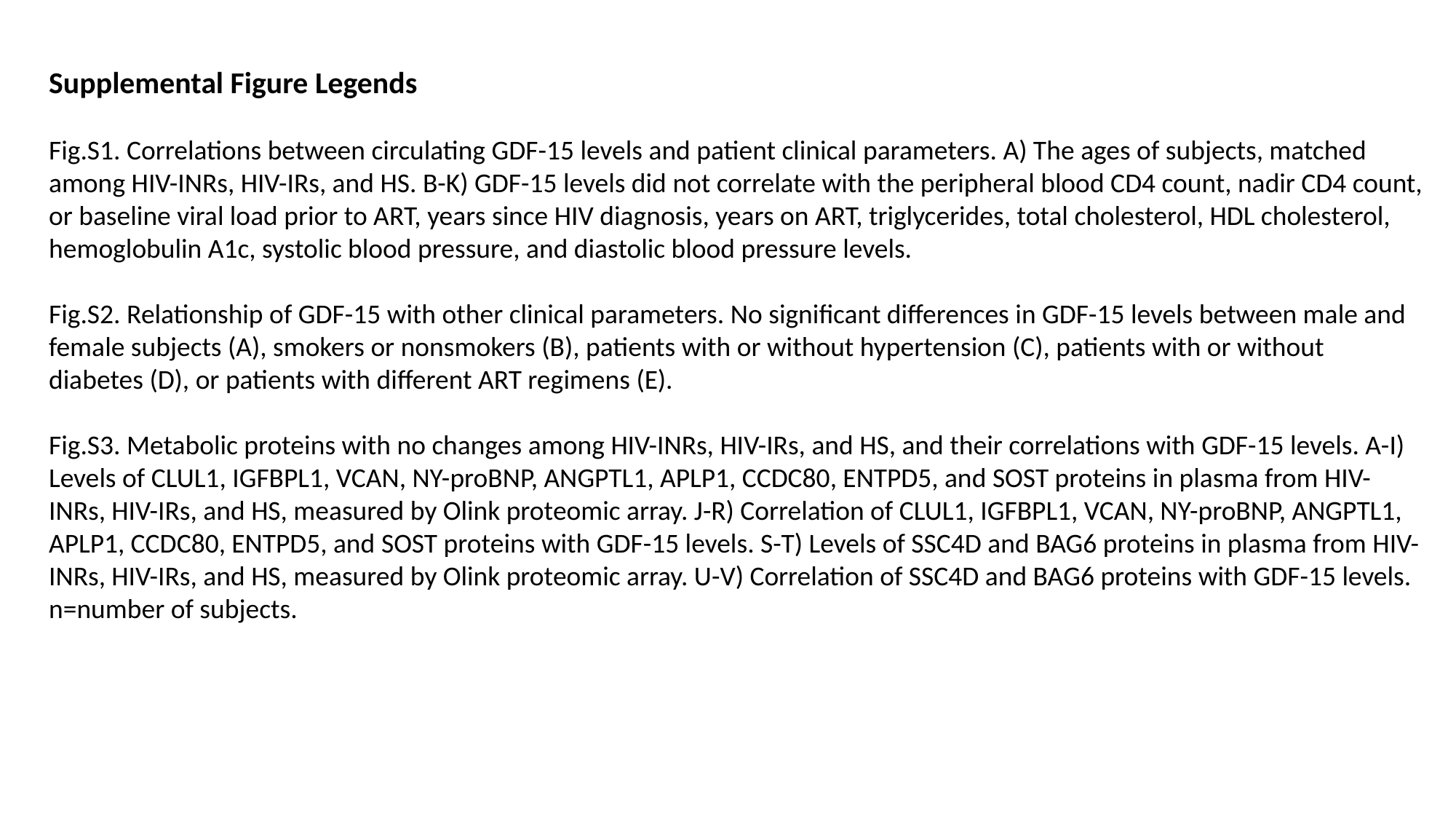

Supplemental Figure Legends
Fig.S1. Correlations between circulating GDF-15 levels and patient clinical parameters. A) The ages of subjects, matched among HIV-INRs, HIV-IRs, and HS. B-K) GDF-15 levels did not correlate with the peripheral blood CD4 count, nadir CD4 count, or baseline viral load prior to ART, years since HIV diagnosis, years on ART, triglycerides, total cholesterol, HDL cholesterol, hemoglobulin A1c, systolic blood pressure, and diastolic blood pressure levels.
Fig.S2. Relationship of GDF-15 with other clinical parameters. No significant differences in GDF-15 levels between male and female subjects (A), smokers or nonsmokers (B), patients with or without hypertension (C), patients with or without diabetes (D), or patients with different ART regimens (E).
Fig.S3. Metabolic proteins with no changes among HIV-INRs, HIV-IRs, and HS, and their correlations with GDF-15 levels. A-I) Levels of CLUL1, IGFBPL1, VCAN, NY-proBNP, ANGPTL1, APLP1, CCDC80, ENTPD5, and SOST proteins in plasma from HIV-INRs, HIV-IRs, and HS, measured by Olink proteomic array. J-R) Correlation of CLUL1, IGFBPL1, VCAN, NY-proBNP, ANGPTL1, APLP1, CCDC80, ENTPD5, and SOST proteins with GDF-15 levels. S-T) Levels of SSC4D and BAG6 proteins in plasma from HIV-INRs, HIV-IRs, and HS, measured by Olink proteomic array. U-V) Correlation of SSC4D and BAG6 proteins with GDF-15 levels. n=number of subjects.
